# Diagnosis Challenges in Adult Leukemia: Insights From a Single-Center Retrospective Study in Qatar (2016-2021)

**DOI:** 10.1177/10732748241275026

**Published:** 2025-03-28

**Authors:** Hesham A. B. Aboelkhir, Yousra El Alaoui, Regina Padmanabhan, Majed Hadid, Adel Elomri, Tanvir Alam, Mohamed Amine Rejeb, Halima EL Omri, Ruba Y. Taha, Hesham Elsabah, Abdelfatteh EL Omri

**Affiliations:** 1 Division of Engineering Management and Decision Sciences, College of Science and Engineering, 534894 Hamad Bin Khalifa University, Doha, Qatar; 2 College of Science and Engineering, 534894 Hamad Bin Khalifa University, Doha, Qatar; 3 Surgical Research Section, Department of Surgery, 36977 Hamad Medical Corporation, Doha, Qatar; 4 Division of Hematology, Department of Medical Oncology, National Center for Cancer Care & Research (NCCCR), 36977 Hamad Medical Corporation (HMC), Doha, Qatar; 5 Vice President for Medical and Health Sciences Office, QU-Health, 61780 Qatar University, Doha, Qatar

**Keywords:** diagnostic delay, hematologic malignancies, leukemia, Qatar, root causes analysis

## Abstract

**Objectives:**

While delays in leukemia detection remain an ongoing challenge in hematologic cancer care, little is known about the factors associated with these delays. This article focuses on identifying the barriers hindering timely diagnosis of leukemia through a cohort analysis (2016-2021) of 220 Acute Myeloid Leukemia (AML), 161 Chronic Myeloid Leukemia (CML), 90 Acute Lymphocytic Leukemia (ALL), and 121 Chronic Lymphocytic Leukemia (CLL) patients in Qatar.

**Methods:**

Of the 592 patients used for the study, subsets were identified and analyzed for delay (423), risk stratification (437), and leukemia stage (282).

**Results:**

There was an increasing trend in leukemia cases, with 32% of patients being diagnosed in the high-risk category. Out of 423 (median delay = 28 days) patients, 45% reported delayed diagnosis (median delay = 44 days). Further analysis of the association of delayed leukemia diagnosis using the univariate χ 2 independence test revealed significant associations to patient referral type, and the presence of certain comorbidities and symptoms.

**Conclusion:**

Significant delays in leukemia diagnosis were identified, though the exact cause remains unclear. These delays can be attributed to factors such as patient, primary care, referral, system, and physician delays. Therefore, further investigation is imperative for improving the detection, diagnosis, and referral processes in hematologic cancers.

## Introduction

Cancer is considered a leading cause of death worldwide, resulting in approximately one in six deaths in 2020.^1^ Moreover, as carcinogens and other risk factors are significantly increasing in high-income countries, worldwide cancer cases are expected to increase by more than 75% by 2030.^1^ Out of all deaths in Qatar, cancer-related deaths constitute 27% of those caused by non-communicable diseases (NCDs).^2^

Unlike easy-to-suspect cancer types, such as breast cancer and melanoma, hematological cancer types are difficult to infer owing to the absence of clear indicators (asymptomatic) and ambiguous symptoms.^3^ Confusion of leukemia symptoms with other less critical diseases can prolong the interval between symptom onset and leukemia diagnosis, resulting in delayed diagnosis. Studies have reported that delays in cancer detection can adversely affect morbidity and mortality and may have physical, psychological, and financial impacts.^4^

Hematological cancers are categorized into three main groups: leukemia, lymphoma, and myeloma. Leukemia comprises four main types: Acute Myeloid Leukemia (AML), Acute Lymphocytic Leukemia (ALL), Chronic Myeloid Leukemia (CML), and Chronic Lymphocytic Leukemia (CLL). Each presents a different pattern of symptoms, spread, and responds to treatment differently, which could result in multiple primary care consultations and potential diagnostic delays.

Descriptive analysis is vital to study the incidence rate of each leukemia type and investigate the association between different factors, such as age, sex, nationality, symptoms, comorbidities, and delay in diagnosis. The information derived from both descriptive and statistical analyses provides decision makers with the necessary insights for strategic health care planning. This includes considerations of health care capacity and the implementation of various interventions to effectively minimize diagnosis delays and waiting times.

While many studies have examined the epidemiology of solid cancers in Qatar,^5–7^ very few have shed light on hematological cancers, namely leukemia diagnostic delay, and its association with demographic and clinical factors. In a study conducted by Maaz et al. (2021),^6^ the median time from symptom onset to diagnosis was 28 days (range 1-845 days) for all central nervous system (CNS) tumors in Qatar. Hence, the primary focus of this work is to close this existing knowledge gap by studying diagnostic delays and the driving factors behind delays in leukemia diagnosis in Qatar.

## Methods

### Data Collection

This is a retrospective study on 592 leukemia cases reported among adults (15-83 years) from 2016 to 2021 at the National Center for Cancer Care and Research NCCCR where data was retrieved from electronic health record (EHR) software Cerner Millennium^®^, and verified by two senior consultants from hematology department. All patients’ details were de-identified using patient ID rather than patients’ names. The IRB committee of the Cancer Center approved this study following hospital policy. The dataset included 381 (64.4%) cases of myeloid leukemia (ML) and 211 (35.6%) cases of lymphocytic leukemia (LL) (Table 1). Each category included the acute (AML and ALL) and chronic (CML and CLL) subtypes. Our study examines all leukemia cases diagnosed in Qatar from 2016 to 2021, given that the National Center for Cancer Care and Research (NCCCR) is the exclusive tertiary hospital for cancer care in the country. The reporting of this study adheres to STROBE guidelines.^8^

**Table 1.** Rate of Leukemia Subtypes Distribution and Socio-Demographic Characteristics of Study Population. Inside the Table, Values are Given in the Following Order: Number of Cases (N) (Percentage of Patients (%) from Total Leukemia Cases and Each Leukemia Subtype), the Total Percentage (TP) for Each 100,000 Populations, and Specific Percentages for Gender, Region, and Age at Diagnosis of Hematological Leukemia Patients in Qatar 2016 -2021. * International Classification of Diseases, 10th Revision (ICD-10).

Description ICD-10 Code *	AML C92.A0	CML C92.10	ALL C91.0	CLL C91.10	Total Leukemia
Years (N (%), total percentage (TP)^a^)					
2016	26 (11.8%), 0.99	17 (10.6%), 0.65	21 (23.3%), 0.80	17 (14%), 0.65	81 (13.7%), 3.09
2017	37 (16.8%), 1.36	38 (23.6%), 1.39	12 (13.3%), 0.44	12 (9.9%), 0.44	99 (16.7%), 3.63
2018	37 (16.8%), 1.34	22 (13.7%), 0.80	16 (17.8%), 0.58	11 (9.1%), 0.40	86 (14.5%), 3.12
2019	35 (15.9%), 1.25	17 (10.6%), 0.61	15 (16.7%), 0.54	23 (19.0%), 0.82	90 (15.2%), 3.22
2020	47 (21.4%), 1.73	25 (15.5%), 0.92	13 (14.4%), 0.48	29 (24.0%), 1.06	114 (19.3%), 4.19
2021	38 (17.3%), 1.42	42 (26.1%), 1.57	13 (14.4%), 0.49	29 (24.0%), 1.09	122 (20.6%), 4.57
Total	220	161	90	121	592
Gender (N (%), gender-specific percentage (GSP)^b^)					
Male M % IR	170 (77.3%), 1.41	124 (77.0%), 1.03	72 (80%), 0.60	101 (83.5%), 0.84	467 (79%), 3.89
Female F % IR	50 (22.7%), 0.91	37 (23.0%), 0.87	18 (20.0%) 0.42	20 (16.5%), 0.47	125 (21%), 2.90
Age (N (%), age-specific percentage (ASP)^c^)					
A1: <20	5 (2.3%), 0.94	--	9 (10.0%), 1.69	--	14 (2.4%), 2.63
A2: [20-24]	7 (3.2%), 0.64	6 (3.7%), 0.55	10 (11.1%), 0.92	--	23 (3.9%), 2.12
A3: [25-65]	187 (85.0%), 1.50	152 (94.4%), 1.22	70 (77.8%), 0.56	93 (76.9%), 0.75	502 (84.8%), 4.03
A4: >65	21 (9.5%), 8.31	3 (1.9%), 1.19	1 (1.1%), 0.40	28 (23.1%), 11.08	53 (9.0%), 20.98
Region (N (%), region-specific percentage (RSP)^d^)					
R1: South Asia	100 (45.5%), 0.93	81 (50.3%), 0.75	50 (55.6%), 0.46	25 (20.7%), 0.23	256 (43.2%), 2.38
R2: MENA	66 (30.0%), 1.28	50 (31.1%), 0.97	18 (20.0%), 0.35	72 (59.5%), 1.40	206 (34.8%), 3.99
R3: East Asia and Pacific	31 (14.1%), 1.80	15 (9.3%), 0.87	11 (12.2%), 0.64	--	57 (9.6%), 3.31
R4: Sub-Saharan Africa	13 (5.9%), 1.38	13 (8.1%), 1.38	9 (10.0%), 0.96	9 (7.4%), 0.96	44 (7.4%), 4.68
R5: Europe and central Asia	8 (3.6%), 2.26	2 (1.2%), 0.56	2 (2.2%), 0.56	11 (9.1%), 3.10	23 (3.89%), 6.48
R6: North America	1 (0.5%), 0.33	--	--	4 (3.3%), 1.33	5 (0.8%), 1.66

Calculations keys.

^a^[(number of new cases diagnosed in the year/Qatar population in that year) *100,000].

^b^[(number of new cases diagnosed from specific gender/the average of Qatar population from this gender from to 2016-2021) * 100,000].

^c^[(number of new cases diagnosed from specific age category/the average of Qatar population from this age category from to 2016-2021) * 100,000].

^d^[(number of new cases diagnosed from specific region category/the average of Qatar population from this region category from to 2016-2021) * 100,000].

### Statistical Analysis on Delays

Clinical and demographic data were collected to assess the risk factors associated with delayed diagnosis in patients with leukemia in Qatar. Demographic parameters included sex, age, and nationality, whereas clinical features included year of diagnosis, referral type, comorbidities, symptoms, date of symptom presentation as reported by the patient, and date of diagnosis.

To identify all potential associations between the diagnostic delay pertaining to leukemia and the aforementioned factors, we used IBM SPSS Statistics 29.0.0.0 to perform univariate analysis using χ 2 independence test. We used the Chi-square test to assess whether there is a significant association between two categorical variables, such as a demographic factor and diagnosis delay. Additionally, we conducted subgroup analyses based on the type of leukemia (AML, ALL, CML, or CLL), considering that different patterns in distinct subtypes might influence the delay in leukemia presentation. In our center, the laboratory turnaround times (TAT) are typically 1 h for urgent orders and 4 h for routine orders. For flow cytometry analysis, TAT ranges from 4 h for urgent orders to 48 h for routine orders.

### Benchmarking

A comparison between the cases reported in Qatar and other countries was conducted based on statistics retrieved from the WHO database and GLOBOCAN 2020 map production.^9^ A comparison between the diagnostic delay reported in Qatar and that reported in other countries was also conducted.

### Other Statistical Analysis

The study categorized the patients into four age groups: <20 (A1), 20-24 (A2), 25-65 (A3), and >65 years (A4). Next, 52 nationalities in the dataset were categorized into seven region-wise groups according to the UNICEF and World Bank regional classification: South Asia (R1), Middle East and North Africa (MENA) (R2), East Asia and Pacific (R3), Sub-Saharan Africa (R4), Europe and Central Asia (R5), North America (R6), and Latin America and Caribbean (R7).^10^

Newly diagnosed cases were analyzed using Total Percentage (TP, number of new leukemia cases in the period/total population in the period), gender-specific percentage (GSP, number of new leukemia cases per gender in the period/total population per gender in the period), Age-Specific Percentage (ASP, number of new leukemia cases per age category in the period/total population per age-category in the period), and Region-Specific Percentage (RSP, number of new leukemia cases per region in the period/total population per region in the period) per 100,000 people per year.

## Results

### Baseline Statistics

Based on our data, AML is regarded as the most common subtype (37% of all leukemia cases), followed by CML (27%), CLL (20%), and ALL (15%). Gender-wise, approximately 79% of all leukemia cases were males; this significantly higher prevalence in males might be due to the current gender distribution in Qatar, where 72%–75% are male.^11^ The GSP per 100,000 also showed that for each leukemia type, the percentage in males was higher than that in females (Table 1). For all leukemias in Qatar, the yearly average GSP was 3.89 for males and around 2.90 for females.

**Figure 1.**
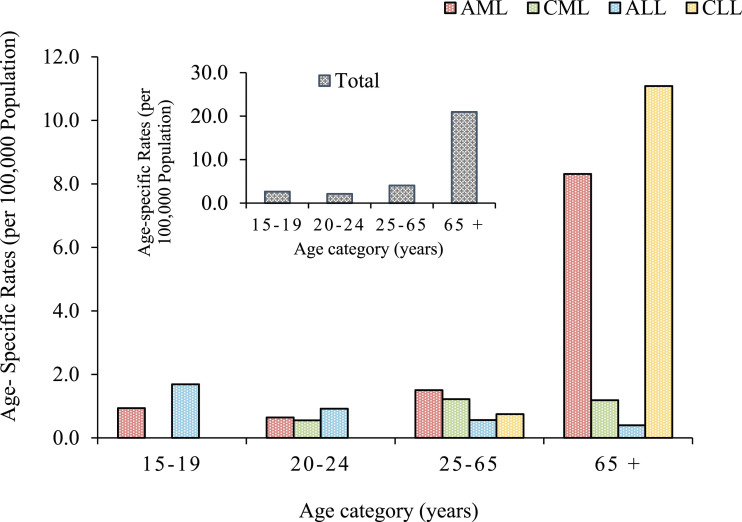
Age-specific rates of hematologic malignancies in Qatar (per 100,000 population).

For CML, AML, CLL, and ALL, the dataset included patients age range (32-83), (22-73), (16-81), and (15-70), and the average age for each subtype was 58.8, 41.3, 42.8, and 33.0, respectively. The overall leukemia ASPs were 2.63, 2.12, 4.03, and 20.98 for the A1 to A4 groups, respectively, with A4 having the highest percentage. The steady increase in ASP with advancing age is attributed to DNA damage and immunosuppression.^12^ Regarding subtypes, for AML and CLL, ASPs were highest for A4, whereas for CML, ASP for A3 (1.22) and A4 (1.19) were closer (Figure 1). However, for ALL, age category A1 (15-19 years) presented the highest ASP, matching the global trends.^13,14^ Next, the RSP was analyzed as per the data of 52 nationalities across seven regions (R1-R7). Overall, leukemia RSPs were highest for R5 (Europe and Central Asia). Subtype-wise RSPs were highest in region R5 for AML and CLL, and in region R4 (Sub-Saharan Africa) for CML and ALL.

### The Trend of Leukemia Types per Year from 2016 to 2021

**Figure 2.**
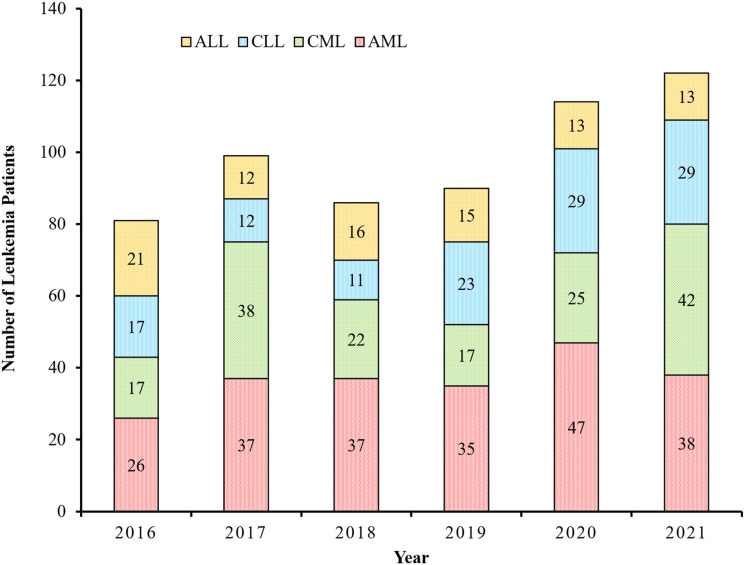
Annual distribution of leukemia subtypes in Qatar (2016-2021).

The annual number of cases for all nationalities for each leukemia type is shown in Figure 2.

While the newly diagnosed percentages of CML, AML, and CLL increased during 2016-2021, ALL showed a decreasing trend during this period (Table 1). Moreover, all leukemias showed a slightly increasing trend which may potentially create a capacity issue and increase diagnostic delays, necessitating more facilities to minimize increased waiting times. Furthermore, such capacity issues and delays in diagnosis could potentially affect the quality of leukemia treatment, reduce patient survival rates, and increase health care costs.

Hence, it is imperative to take actions aligned with the strategic plan of Qatar’s 2030 vision, which recommends accounting for any forecasted increase in health care demand by expanding the government sector, supporting the private health care sector, promoting cooperation and coordination between both sectors, and improving accessibility to health care services.

### Leukemia Distribution as per WHO Classification

**Figure 3.**
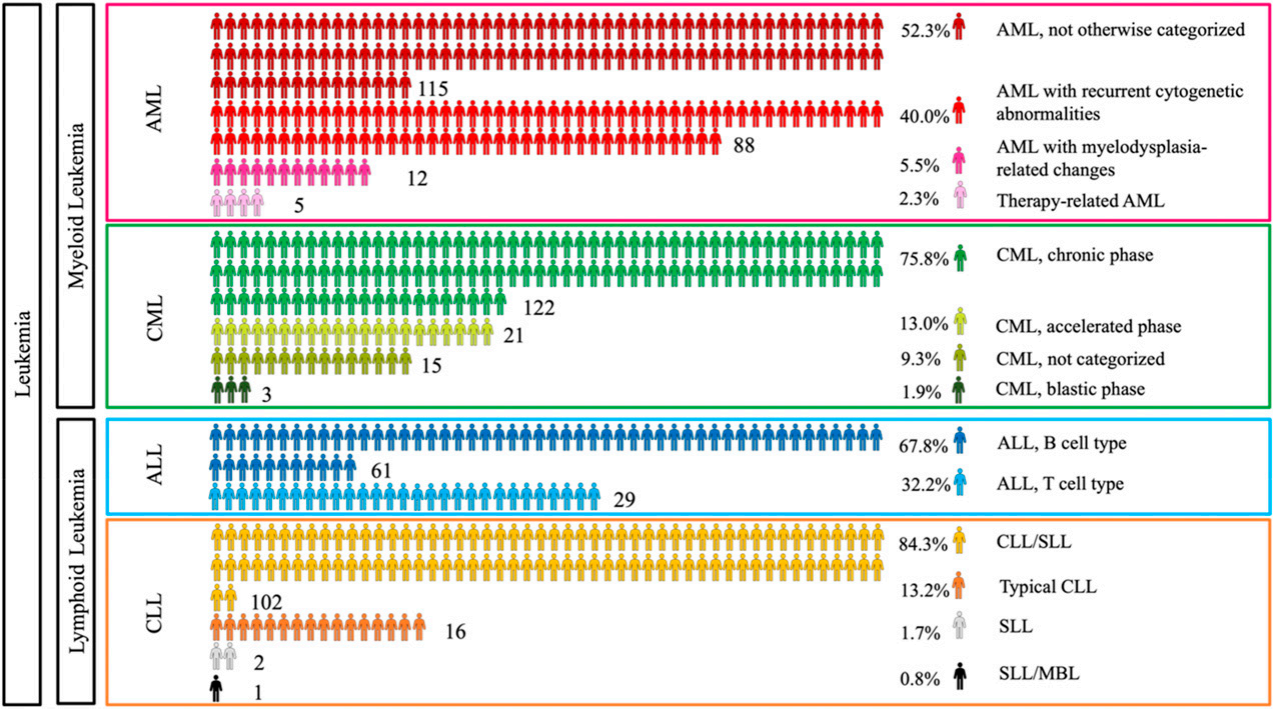
Classification of leukemia cases diagnosed in Qatar from 2016-2021 based on WHO Categorization ICD-O-3.2 fifth version (2022).

To analyze leukemia distribution, the WHO classification of hematological cancers and the International Classification of Diseases for Oncology (ICD-O-3) fifth version coding ^14^ were used, where the distribution of myeloid and lymphoid leukemia and their subtypes was visualized. AML was categorized into four subtypes: AML not otherwise categorized 115 (52.3%) AML with recurrent cytogenetic abnormalities 88 (40.0%) such as acute promyelocytic leukemia (APL) 44 (20.0%) AML with myelodysplasia-related changes 12 (5.5%), and therapy-related AML 5 (2.3%) (Figure 3).

Similarly, CML was categorized into three phases: chronic phase, 122 (75.8%), accelerated phase, 21 (13.0%), and blastic phase, 3 (1.9%). Notably, 15 CML patients (9.3%) did not undergo a bone marrow aspirate and were therefore not categorized. ALL was categorized based on their phenotype into two main subcategories: B cell lymphoblastic 61 (67.8%) and T cell lymphoblastic 29 (32.2%). Even though cytogenetic based classification was used for clinical decision making this study did not use that data.

Finally, CLL was categorized into four subcategories: CLL with small lymphocytic lymphoma CLL/SLL, which was the most common in our study 102 (84.3%), typical CLL 16 (13.2%), small lymphocytic lymphoma SLL 2 (1.7%), and atypical small lymphocytic lymphoma/monoclonal B-cell lymphocytosis SLL/MBL 1 (0.8%).

### Risk Stratification and Staging

Typically, doctors rely on cytogenetic findings at diagnosis to classify patients with leukemia. This classification is important because there are differences in disease prognosis and treatment options for various cytogenetic abnormalities. The three prognostic categories of AML, CML, and ALL. Based on cytogenetic findings, namely favorable risk (low), intermediate risk, and adverse risk (high), are presented in Table 2. The CLL risk stratification data was not available.

**Table 2.** Risk Stratification Distribution Within Leukemia Patients in Qatar From 2016-2021. * in this table, 34 AML cases were excluded Because their data were incomplete, and CLL risk stratification data were not available.

Risk Stratification N (%) from Each Leukemia Subtype	AML * (N = 186) 42.6%	CML (N = 161) 36.8%	ALL (N = 90) 20.6%	Total (437)
High risk	64 (34.4%)	35 (21.7%)	42 (46.7%)	141 (32%)
Intermediate risk	54 (29.0%)	62 (38.5%)	5 (5.6%)	121 (28%)
Low, favorable, or standard risk	36 (19.4%)	59 (36.6%)	10 (11.1%)	105 (24%)
Not categorized or not documented	32 (17.2%)	5 (3.1%)	33 (36.7%)	70 (16%)

Considering all leukemia cases reported in Qatar, 32% were in the high-risk category, and for acute categories such as AML and ALL, 34.4% and 46.7% were in high-risk side. In the case of CML, 38.5% belonged to the intermediate-risk category (Table 2). In China, (23.5%) AML patients were classified into adverse risk category.^15^ While in Dana-Farber Cancer Institute, 45% of reported ALL cases were in high-risk category.^16^ Further analysis is warranted to identify whether known risk factors for leukemia, including environmental and population-specific genetic factors, explain the high number of diagnoses in the high-risk category. Conducting these analyses may uncover previously unknown factors related to leukemia.

Apart from the classification predicated on the risk prognosis, patients with chronic leukemia are also categorized based on cell counts. For instance, based on the percentage of blast in the bone marrow at time of diagnosis, CML patients are classified into chronic, accelerated, and blast phases (Table 3). The chronic phase of CML is usually the earliest and most common one. Contrary to the chronic phase, patients in the accelerated phase do not respond favorably or efficiently to treatment.^17^

**Table 3.** Stages or Phases Within Chronic Leukemia Patients in Qatar From 2016-2021.

Features	CML (N = 161) 29%	Features	CLL (N = 121) 22%
Chronic leukemia phases and staging (CML, CLL)			
Chronic phase (<10% blasts)	122 (75.8%)	A binet stage (<3 areas of lymphadenopathy, No anemia or thrombocytopenia)	26 (21.5%)
Accelerated phase (10%–19% blasts)	21 (13.0%)	B binet stage (>3 areas of lymphadenopathy, No anemia or thrombocytopenia)	36 (29.8%)
Blastic phase (≥20% blasts)	3 (1.9%)	C binet stage (>3 areas of lymphadenopathy anemia or thrombocytopenia)	24 (19.8%)
Not categorized or not documented	15 (9.3%)	Not categorized or not documented	35 (28.9%)

Another type of staging is the Binet staging system for CLL, which mainly depends on the presence of anemia, thrombocytopenia, and the number of lymphadenopathies. For instance, in Mohammed, S et al, the median patient survival rate was significantly higher among patients in Binet stage A than among those in the Binet stages B or C.^18^ A comparison between CLL Binet staging at diagnosis in Qatar as per our data and that reported in De Moraes Hungria,V.T et al. for Latin America^19^ revealed that the high-risk Binet stage C rate in Qatar (19.8%) exceeded that in Latin American countries (11.8%) (Panama, Chile, Argentina, Colombia, Mexico, and Brazil). Conversely, the low-risk Binet stage A rate in Latin America corresponds to 40% of the total cases, compared to only 21.5% in Qatar.

### Delay in Diagnosis

Several studies have focused on identifying different factors that hinder the timely diagnosis of leukemia in patients. However, no standard definition of delay in diagnosis has been established.^20–22^ In^21^, the term “delay” is used to define the time interval in days elapsed between the onset of symptoms and diagnosis confirmation. By consensus, a delayed leukemia diagnosis is defined as a period exceeding one month from symptom onset to a confirmed diagnosis.^22^ The objective of the present study was to analyze existing delays and determine the factors influencing these delays in patients with leukemia in Qatar.

**Figure 4.**
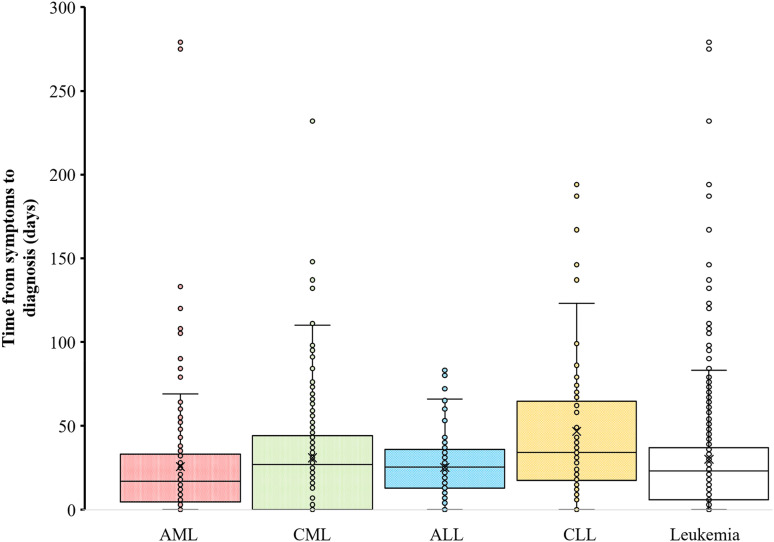
Temporal Analysis: Time from Symptoms to Diagnosis Distribution (in Days) in hematological malignancies in Qatar (2016-2021).

According to Figure 4, chronic leukemia patients (CLL and CML) seem to experience a longer time interval between symptom onset and diagnosis than acute leukemia patients, aligning with the slow development of chronic malignancies. The rapid growth of acute leukemia reveals its symptoms, prompting patients to seek prompt medical advice.

Out of 592 patients, only 558 patients were included in the study for symptoms, comorbidities and referral systems, as specific details about symptoms, comorbidities, and referral systems were not available for the rest of the patients. Of the 558 patients, 436 were symptomatic and 122 were asymptomatic and did not exhibit any symptoms before their actual diagnosis dates. Out of the 436 symptomatic patients, only 423 were included in the delay analysis due to the lack of specific details about delays for the remaining patients. Higher rates of asymptomatic cases were found in patients with chronic leukemia (CLL 47.9%, CML 30.4%) compared to those with acute leukemia (Table S1). We studied cases that showed ≥30 days delay from symptoms to diagnosis individually to determine the latent cause of delay (Table 4).

**Table 4.** Diagnostic Delay and Referral Patterns Among Leukemia Patients in Qatar From 2016-2021.

Description	AML (N = 186) 33%	CML (N = 161) 29%	ALL (N = 90) 16%	CLL (N = 121) 22%	Total (558)
Time from symptoms to diagnosis (days)					
Documented	167 (89.8%)	109 (67.7%)	84 (96.7%)	63 (53.7%)	423 (77%)
Min-max (μ, σ)	1-279 (28.6, 37.1)	2-232 (45.7, 34.5)	1-83 (26.0, 17.6)	0-194 (64.7, 43.7)	0-279 (35.2, 35.6)
Median (25-75 percentile range)	20 (7-35)	35 (26.5-56)	26 (13-36)	34 (17.5-64.5)	28 (13.25-41)
Referral center					
Emergency	139 (74.7%)	63 (39.1%)	52 (57.8%)	1 (0.8%)	255 (46%)
GH^a^ outpatient	8 (4.3%)	91 (56.5%)	3 (3.3%)	--	102 (18%)
GH^a^ inpatient	26 (14.0%)	7 (4.3%)	35 (38.9%)	4 (3.3%)	72 (13%)
Partnered cancer center^b^	12 (6.5%)	--	--	113 (93.4%)	125 (22%)
Others (primary care, private sector, and other government hospitals)	1 (0.5%)	--	--	3 (2.5%)	4 (1%)

^a^GH: General Hospital.

^b^NCCCR: The National Center for Cancer Care and Research (NCCCR).

### Potential Diagnosis Delays and Their Association with Epidemiological Variables

Out of the 558 patients, delay analysis was conducted on 423 cases. Among these, 192 patients (45%) experienced delayed diagnosis. For all 423 documented cases, the median time from symptom onset to diagnosis was 28 days (range: 0-279 days). However, for the 192 patients with delayed diagnosis (time to diagnosis ≥30 days), the median time was 44 days. Most of the reported symptoms among leukemia patients were common, with only 18% of the overall cases showing organ or lymph node swelling, regarded as the most distinctive indicator of leukemia.

In our univariate analysis, demographic variables such as age, sex, and nationality were not associated with delayed diagnosis. The analysis also showed a significant difference in diagnostic delay between patients who were directly referred to a hematologist upon symptom onset and those who underwent multiple consultations before being referred to a specialist (*P*-*value* = 0.042).

Moreover, leukemia patients exhibiting the following signs and symptoms: fever, sweating, and flu-like symptoms (S1), cough, shortness of breath and respiratory problems (S4), anorexia and weight loss (S5) (*P*-*value* <0.015 each), organ or lymph node swelling (S6), and bleeding/bruising (S7) (*P*-*value* <0.001 each) were associated with diagnostic delay (Table S1). The univariate chi-square test revealed whether there is a significant association or not; to know the nature of association (delay is more likely or less likely), we created contingency table and examined the proportions of delayed vs not delayed (<30 days) patients in each category. Out of the significantly associated symptoms (S1, S4, S5, S6, S7, S11) and comorbidities (C13, C18, C19, C22), delay is more likely for variables S5, S6, and C22 for which majority of patients reported diagnostic delay (≥30 days), however, time to diagnosis of major proportion of patients who had other symptoms and comorbidities (S1, S4, S7, S11, C13, C18, C19) were within 30 days. The average of all reported time to diagnosis for patients with S5 (Anorexia and weight loss, S6 (Organs or lymph node swelling), and C22 (other comorbidities) were 43.9, 45.8, 33.4 days, respectively. Symptoms such as S1 (Fever, sweating, and flu-like symptoms) and S7 (Bleeding) were associated with less likelihood of late diagnosis, average time to diagnosis being 29.8 (1-275) and 28.6 (2-132) days, respectively. This may be expected since patients presenting with these symptoms are typically referred to hematologists. While other symptoms such as localized and generalized body pain, generalized fatigue, weakness and malaise, nausea and vomiting, sore throat, tonsillitis, pharyngitis, mouth ulcers, and dizziness, did not show a significant association with diagnostic delay (Table S1).

Moreover, many showed a set of unhealthy patterns, such as smoking, obesity, and alcoholism, representing 4.8%, 3.9%, and 0.7%, respectively, of the overall patients included in the study. The number of comorbidities per patient was analyzed, with hypertension being the most common comorbidity in 117 patients (21% of all patients), followed by diabetes mellitus DM (19.7%). In addition, 20 patients were diagnosed with other types of cancer, including breast cancer (4 out of 20), thyroid cancer (3 out of 20), prostate cancer (3 out of 20), and various other cancers (10 out of 20). When patients have other comorbidities, they may attribute some symptoms to their existing conditions rather than a new disease, which can delay seeking medical care. Conversely, frequent visits to doctors for existing conditions (comorbidities) increase the chance of incidental leukemia diagnosis. In our study, comorbidities such as hypertension (C1), Type II Diabetes Mellitus (C2), respiratory diseases (C3), lipid profile disorder (C4), past surgical procedures (C5), cardiac and arterial diseases (C6), liver and spleen diseases (C7), kidney and urinary tract diseases (C8), other types of cancer (C9), smoking (C10), thyroid gland disorders (C11), infectious diseases and inflammations (C12), obesity (C14), benign tumors and fibrosis (C15), arthritis (C16), stroke and brain disorders (C17), gout (C20), and alcohol consumption (C21) did not show a significant association with diagnostic delay (Table S1). Similarly, our results highlight the existence of a statistically significant association between the year of diagnosis and diagnostic delay. Even though the year of analysis is a significant variable, the nature of the association remains unclear. The proportions of patients with delayed diagnosis (time to diagnose ≥30 days) were 53% in 2016, 57% in 2017, 42% in 2018, 31% in 2019, 34% in 2020, and 53% in 2021. The average times to diagnosis for these years were 37.9, 42.6, 37.9, 27.7, 27.2, and 38.7 days, respectively.

## Discussion

### Clinical Significance and Recommendations

Previous studies have analyzed different scenarios and tested several variables to identify the factors influencing diagnostic delays.^21,22^ Table 5 presents a comparison between our study and similar recent studies that measured delay in diagnosis in the UK, Brazil, China, Rwanda, and India.^23–28^ As detailed in Table 5, the median time to diagnosis reported in Qatar (28 days) is comparable to findings from studies in India (32 days), Rwanda (32 days), Brazil (30 days), and China (21 days). In our study, a significant association was identified between leukemia diagnosis delay and the patient’s referral type. This finding aligns with a study involving hematologic cancer patients in Lithuania,^20^ which demonstrated that seeing more specialists before diagnosis significantly prolongs diagnostic intervals (*P*-value = 0.022). Similarly, a study in Brazil concluded that AML and ALL patients who visited an outpatient clinic were more likely to be diagnosed late compared to those assessed in emergencies (*P-value* = 0.04).^29^ A possible explanation could be that the more time it takes for patients to be referred to specialists, the longer it takes for their diagnosis to be confirmed.

**Table 5.** Comparison Between Our Study and Recent Similar Literature.

Author (year) [Ref]	Cancer	Age	Region	Study years	Methodology	Median Time to Diagnosis
Din et al^23^ (2015)	15 cancers	>=40	UK	2007-2010	Analysis of data from the UK clinical practice research datalink	Leukemias diagnostic interval (median 102 days)
Guzman C et al (2021)^24^	ALL, AML	0-14	Brazil and Colombia	2000-2020	Retrospective study	Columbia: ALL 7days, Brazil: ALL 18 days, AML 13 days (median)
Ribeiro et al^25^ (2021)	Pediatric cancer hematologic tumors 49%	0-18	Brazil	2013-2016	Prospective observational study, 146 pediatric cancer patients	The median time to diagnosis for AML and ALL was 30 days
Dai et al^26^ (2021)	ALL	0-14	China	2011-2015	Retrospective cohort study, 419 patients	The median time to diagnosis 21 days
Morgan et al (2022)^27^	CML	8-81	Rwanda	2009-2018	Retrospective study, 99 patients	The median time to diagnosis was 32 days
Lashkari et al^28^ (2021)	ALL, AML	0-14	India	2014-2016	Retrospective study, 111 cases	Median ALL 33 days AML 32 days
This article (2023)	AML, ALL, CLL, CML	15-83	Qatar	2016-2021	Retrospective study, 592 leukemia patients	Median 28 (13.25-41) days Mean 35.2 SD 35.6 days

Furthermore, our present work suggests an association with the year of diagnosis, which might be linked to the increasing prevalence during the study period due to recent population growth and immigration to the state of Qatar. As a result, the sudden additional burden on the health care system may have contributed to diagnostic delays.

A study carried out in Brazil, suggested that delay is mainly driven by an exhibition of symptoms such as bone or joint pain,^29^ however, in our study S2 (localized and generalized body pain) was reported in 180 cases and was not associated with delay. This may be due to similar proportion of delayed (78 cases with average delay = 60.2 days) and not delayed (102 cases with average delay 16 days) patients. Another study in India assessed different social factors and their potential associations with delays in diagnostic intervals in pediatric hematological patients.^28^ The latter study found that paternal age of less than 30 years and less than 8 years of parental education highly affected a leukemia patient’s time to diagnosis.

In the context of comorbidities, the findings suggest that blood diseases and anemia (C13), autoimmune diseases and allergies (C18), psychiatric disorders and neurological diseases (C19), are significantly associated with delay in leukemia diagnosis. Majority of patients with C13, C18, and C19 were diagnosed within 30 days, indicating less likelihood of late diagnosis. However, a study conducted in Lithuania, stressed the link between patients exhibiting high anxiety and depression levels and a significant increase in their time to diagnosis.

The presence of common symptoms and comorbidities among leukemia patients can play a significant role in delaying the diagnostic process, as these signs could be mistaken for normal signs, leading physicians to either ignore the symptoms or attribute them to the present comorbidities. With advancements in artificial intelligence (AI) and data analytics in the field of leukemia detection, incorporating AI-based smart diagnostic devices can significantly reduce delays by examining the intricate details of symptoms, comorbidities, and other routine test results.^30^

The comparison between Qatar’s incidence rate and that of other countries, using data retrieved from the WHO database and the GLOBACAN 2020 map production, showed that Qatar exhibited the second lowest (3.2) crude incidence rate (CIR) compared to other GCC countries, such as Saudi Arabia (4.8), Kuwait (4.8), Oman (4.2), and Bahrain (3.3), with the United Arab Emirates having the lowest rate (2.8).^31^ Regionally, Iran had the highest CIR (7.3), followed by Lebanon (7) and Syria (5.8). Globally, the United States is ranked first, with a rate of 18.5, followed by France (18), Australia (17.6), Canada (17.5), and the United Kingdom (16.2).

Qatar has the second lowest leukemia incidence rate (IR) in the Gulf region, with 3.2 cases per 100,000 population. However, the number of cases has increased from 2016 to 2021, and the anticipated rise in these numbers over time necessitates an assessment of whether existing health care resources (experts, diagnostics, and tools) can effectively address this projected surge in patient numbers. Although the WHO database and global cancer registries are regarded as good references for incidence rates, very few studies have shed light on leukemia diagnostic delays. Additionally, studies focusing on hematological cancer diagnosis were significantly lower compared to other oncological types. This gap in the literature might be due to the challenging aspects of current leukemia diagnosis techniques and the complexity of hematological cancer symptomatology.

Although early detection has been proven to significantly enhance the quality of treatment and survival rates for leukemia patients, as shown in Table 5, there is a significant delay in leukemia diagnosis; hence, further investigation and intervention are imperative in the field of hematology detection.^32^ By studying the risk stratification for total leukemia, most of the cases diagnosed were in the high-risk category (32%).

Statistical analysis showed that demographic variables, such as age, sex, and nationality, were not associated with delayed diagnosis. This is a good indicator of the quality and fairness of health care access and could also be a result of the free model of Qatar’s health care system and the nature of treatment provided for cancer patients in Qatar.

The analysis showed that patients who were directly referred to a hematologist experienced significantly shorter delays than those who underwent multiple consultations. The percentage of eligible direct referrals may be enhanced through training programs for general physicians to identify the most likely cancer suspects according to the local and international cancer referral guidelines.

We also review potential interventions, initiatives and projects aimed at optimizing hematological malignancy detection based on literature (Table 6). Five out of 8 initiatives listed in Table 6 were found to be effective after implementation in reducing blood cancer diagnosis delay in various countries.^33–40^

**Table 6.** Initiatives to Minimize Delay in Hematological Cancer Diagnosis.

Reference	Country (Year)	Cancer Type	Nature of Symptoms	Initiative	Effectiveness	Observations
^33^	United States of America (2020)	Lymphoma and nine other cancer types	Non-specific	DETECT-A (detecting cancers earlier through elective mutation-based blood collection and Testing)	Effective	These data demonstrate that multi-cancer blood testing can detect cancers in the early stages
						*Leukemias excluded due to difficulties of being detected through blood test screening
^34^	United Kingdom (2014)	Myeloma and nine other cancer types	Specific and non-Specific	2005 NICE referral	Effective	Significant reduction in mean diagnostic interval from 2001-2002 to 2007-2008
				Guidelines		
^35^	Ireland (2016)	Hairy cell leukemia (HCL)	Specific and non-Specific	Audit the compliance with british committee for standards in hematology (BCSH)	Not identified	Areas of improvement in the diagnosis and assessments of treatment were highlighted in response to HCL patients.
^41^	United Kingdom (2021)	All types	Specific and non-specific	Examine the use of the 2-week referral pathway in practice and its relation to diagnosis and waiting time	Not identified	Higher cancer detection via 2WW referrals was associated with larger practices and those with younger GPs
^37^	Taiwan (2016)	AML	Specific and non-specific	Proactive consultation by a clinical pathologist (PCCP)	Effective	PCCP minimize diagnostic delay, particularly in CLL, MM, and MDS/MPN cases, via shortening the help-seeking to diagnostic interval
		ALL, CML				
		MM, and MDS/MPN				
^38^	United Kingdom (2017)	Leukemia, lymphoma, multiple myeloma, and other 17 cancer types	Specific and non-specific	2015 National	Effective	Determine quality methods improvement activity and determine avoidable delay and to what factors it was attributable
				Institute for health and care excellence guidance on management and referral of suspected cancer		
^39^	Botswana (2018)	All types of cancer	Specific	Training program for the trainee in primary care	Effective	Aiming to reduce health systems delays in cancer diagnosis in sub-Saharan Africa. The training achieved district-wide participation in 176 trainees who reported high satisfaction with the training program
^40^	Denmark (2015)	Serious non-specific symptoms and signs of cancer 10% hematopoietic tissue cancer	Specific	Implementation on patients with serious non-specific symptoms and signs of cancer pathway (NSSC-CPP)	Not identified	Relying on GPs gut feeling, 16.2 % of all patients referred through the Danish NSSC-CPP because of non-specific serious symptoms had cancer
						Diagnosis of different types

The findings suggest that a change in the health care policy, which includes strict adoption of existing or revamped referral guidelines, regular auditing in hospitals for compliance with the standards, providing the option of proactive consultations with an expert, and incorporating training programs at the primary care level, resulted in positive outcomes in terms of reduced delays in various hospitals.^33,34,37–40^ Interventions such as training programs for physicians and revising referral guidelines are crucial for reducing doctor delays.

On the other hand, population awareness and improved health care access could help minimize different types of delays in cancer patient pathways. Although AI-driven models and decision support systems are highly recommended to reduce diagnosis delays, such novel strategies are yet to be practically integrated into hospital settings.

Although our study was comprehensive, it has some limitations that need to be addressed, as it focuses solely on leukemia in adult patients. Moreover, the current data did not provide the first general physician consultation dates and follow-ups, which could also allow us to track different types of delays in the cancer pathway, such as patient delay (time between the onset of symptoms and the request for primary care assistance), doctor delay (time from the first consultation to diagnosis). Even though this study captures all cases reported in Qatar, there can be referral bias, presentation bias, other patterns of care seeking that may influence the number of reported cases. Another limitation of our study is we used univariate chi-square test of independence, which tells whether there is a statistically significant association between two categorical variables (example, the year of diagnosis and the delay in diagnosis), but it does not directly indicate the direction or nature of the association (ie, whether delays are increasing or decreasing over the years). In addition, the absence of data such as follow-up, treatment outcomes, and mortality data hindered the calculation of prevalence rates and correlation between diagnosis delay and treatment outcomes, which could be some potential directions for future work. A pivotal direction for future work could be studying the factors influencing delay and treatment outcomes associated with diagnosing APL, a medical emergency. As per our data, 11 out of 44 APL patients report delayed diagnosis (time to diagnose ≥30 days), the average delay for all 44 APL cases is 22.3 days (1-133 days).

## Conclusion

To the best of our knowledge, this is the first study focusing on delays in leukemia diagnosis in Qatar, and our findings are comparable to those from centers in other countries such as India, Rwanda, Brazil, and China. Our analysis indicated that symptoms such as anorexia, weight loss, and organ or lymph node swelling were associated with late diagnosis, while symptoms like fever, sweating, cough, respiratory problems, bleeding, and bruising were associated with diagnosis within 30 days. For future research, we recommend conducting a multivariate analysis on a larger sample size to better understand the factors contributing to diagnostic delays.

## Supplemental Material

Supplemental Material - Diagnosis Challenges in Adult Leukemia: Insights From a Single-Center Retrospective Study in Qatar (2016-2021) Supplemental Material for Diagnosis Challenges in Adult Leukemia: Insights From a Single-Center Retrospective Study in Qatar (2016-2021) by Hesham A. B. Aboelkhir, Yousra EL Alaoui, Regina Padmanabhan, Majed Hadid Adel Elomri, Tanvir Alam, Mohamed Amine Rejeb , Halima EL Omri, Ruba Y. Taha, Hesham Elsabah, and Abdelfatteh EL Omri in Cancer Control

## Data Availability

Research data supporting this publication are available under reasonable request to the corresponding author.*

## Appendix

### List of abbreviations

AI: Artificial Intelligence
ALL: Acute Lymphocytic Leukemia
AML: Acute Myeloid Leukemia
APL: Acute Promyelocytic Leukemia
ASP: Age-Specific Percentage
CIR: Crude Incidence Rate
CLL: Chronic Lymphocytic Leukemia
CLL/SLL: CLL with Small Lymphocytic Lymphoma
CML: Chronic Myeloid Leukemia
EHR: Electronic Health Record
GCC: Gulf Cooperation Council
GSP: Gender-Specific Percentage
ICD-O-3: International Classification of Diseases for Oncology version 3
IR: Incidence Rate
MENA: Middle East and North Africa
NCCCR: National Center for Cancer Care and Research
NCDs: Non-Communicable diseases
RSP: Region-Specific Percentage
SLL: Small Lymphocytic Lymphoma
SLL/MBL: SLL with monoclonal B-cell Lymphocytosis
TAT: Turnaround Time
TP: Total Percentage
WHO: World Health Organization
