# Supplementary material for: Diagnosis Challenges in Adult Leukemia: Insights From a Single-Center Retrospective Study in Qatar (2016-2021)
Source: Cancer Control. 2025 Mar 28;32:10732748241275026. doi: 10.1177/10732748241275026 (PMC11954518; doi:10.1177/10732748241275026)
Supplement: Supplemental Material - Diagnosis Challenges in Adult Leukemia: Insights From a Single-Center Retrospective Study in Qatar (2016-2021) [file sj-pdf-1-ccx-10.1177_10732748241275026.pdf]

Table 1:Symptoms and comorbidity distribution between leukemia types

Description	AML (N=186) 33%	CML (N=161) 29%	ALL (N=90) 16%	CLL (N=121) 22%	Total (558)
**Symptoms**					
Asymptomatic	9 (4.8%)	49 (30.4%)	6 (6.7%)	58 (47.9%)	122 (22%)
Symptomatic	177 (95.2%)	112 (69.6%)	84 (93.3%)	63 (52.1%)	436 (78%)
S1: Fever, sweating, and flu-like symptoms	105 (56.5%)	26 (16.2%)	47 (52.2%)	9 (7.4%)	187 (34%)
S2: Localized and generalized body pain	65 (34.9%)	57 (35.4%)	39 (43.3%)	24 (19.8%)	185 (33%)
S3: Generalized fatigue, weakness, and malaise	72 (38.7%)	34 (21.1%)	25 (27.8%)	9 (7.4%)	140 (25%)
S4: Cough, shortness of breath, and respiratory-related symptoms	69 (37.1%)	21 (13.0%)	34 (37.8%)	14n (11.6%)	138 (25%)
S5: Anorexia and weight loss	42 (22.6%)	41(25.5%)	17 (18.9%)	13 (10.7%)	113 (20%)
S6: Organs or lymph node swelling	26 (14.0%)	29 (18.0%)	10 (11.1%)	38 (31.4%)	103 (18%)
S7: Bleeding	52 (28.0%)	4 (2.5%)	17 (18.9%)	5 (4.1%)	78 (14%)
S8: Nausea and vomiting	20 (10.8%)	13 (8.1%)	14 (15.6%)	--	47 (8%)
S9: Sore throat, tonsillitis, pharyngitis, and mouth ulcers	24 (12.9%)	3 (1.9%)	6 (6.7%)	2 (1.7%)	35 (6%)
S10: Dizziness	17 (9.1%)	7 (4.4%)	14 (15.6%)	2 (1.7%)	40 (7%)
S11: Others	78 (41.9%)	56 (34.8%)	35 (38.9%)	22 (18.2%)	191 34%)
**Comorbidity (Number and Type)**					
**Number of comorbidities per patient**					
0 no comorbidity	94 (50.5%)	80 (49.7%)	52 (57.8%)	24 (19.8%)	250 (44.8%)
1	25 (13.4%)	35 (21.7%)	18 (20.0%)	23 (19.0%)	101 (18.1%)
2	23 (12.4%)	23 (14.3%)	11 (12.2%)	27 (22.3%)	84 (15.1%)
3	24 (12.9%)	10 (6.2%)	3 (3.3%)	24 (19.8%)	61 (10.9%)
4+ four and more comorbidities	20 (10.8%)	13 (8.1%)	6 (6.7%)	23 (19.0%)	62 (11.1%)
**Type of Comorbidity**					
C1 Hypertension HTN	37 (19.9%)	28 (17.4%)	6 (6.7%)	46 (38.0%)	117 (21.0%)
C2 Diabetes Miletus DM Type II	36 (19.4%)	27 (16.8%)	8 (8.9%)	39 (32.2%)	110 (19.7%)
C3 Respiratory diseases	14 (7.5%)	12 (7.5%)	8 (8.9%)	19 (15.7%)	53 (9.5%)
C4 Lipid profile disorder	8 (4.3%)	13 (8.1%)	4 (4.4%)	24 (19.8%)	49 (8.8%)
C5 Past surgical procedure	18 (9.7%)	10 (6.2%)	9 (10.0%)	7 (5.8%)	44 (7.9%)
C6 Cardiac and Arteries diseases CAD	11 (5.9%)	5 (3.1%)	4 (4.4%)	14 (11.6%)	34 (6.1%)
C7 Liver and Spleen diseases	11 (5.9%)	6 (3.7%)	5 (5.6%)	11 (9.1%)	33 (5.9%)
C8 Kidney and Urinary tract diseases KD	8 (4.3%)	5 (3.1%)	2 (2.2%)	13 (10.7%)	28 (5.0%)
C9 Previous Cancer	7 (3.8%)	12 (7.5%)	2 (2.2%)	6 (5.0%)	27 (4.8%
C10 Smoking	4 (2.2%)	8 (5.0%)	4 (4.4%)	11 (9.1%)	27 (4.8%)
C11 Thyroid gland disorder	10 (5.4%)	6 (3.7%)	1 (1.1%)	10 (8.3%)	27 (4.8%)
C12 Infectious diseases and Inflammations	14 (7.5%)	4 (2.5%)	4 (4.4%)	5 (4.1%)	27 (4.8%)
C13 Blood diseases and Anemia	6 (3.2%)	12 (7.5%)	7 (7.8%)	5 (4.1%)	23 (4.1%)
C14 Obesity	3 (1.6%)	7 (4.3%)	3 (3.3%)	9 (7.4%)	22 (3.9%)

C15 Benign tumors and Fibrosis	7 (3.8%)	2 (1.2%)	2 (2.2%)	7 (5.8%)	18 (3.2%)
C16 Arthritis	6 (3.2%)	4 (2.5%)	1 (1.1%)	5 (4.1%)	16 (2.9%)
C17 Stroke and Brain disorders	8 (4.3%)	1 (0.6%)	1 (1.1%)	4 (3.3%)	14 (2.5%)
C18 Autoimmune diseases and Allergies	8 (4.3%)	3 (1.9%)	--	2 (1.7%)	13 (2.3%)
C19 Psychiatric disorder and Neurology diseases	3 (1.6%)	3 (1.9%)	1 (1.1%)	4 (3.3%)	11 (2.0%)
C20 Gout	4 (2.2%)	3 (1.9%)	--	1 (0.8%)	8 (1.4%)
C21 Alcohol drinker	1 (0.5%)	2 (1.2%)	--	1 (0.8%)	4 (0.7%)
C22 Others	13 (7.0%)	16 (9.9%)	3 (3.3%)	8 (6.6%)	40 (7.2%)
